# A 16S rRNA Gene and Draft Genome Database for the Murine Oral Bacterial Community

**DOI:** 10.1128/mSystems.01222-20

**Published:** 2021-02-09

**Authors:** Susan Joseph, Joseph Aduse-Opoku, Ahmed Hashim, Eveliina Hanski, Ricarda Streich, Sarah C. L. Knowles, Amy B. Pedersen, William G. Wade, Michael A. Curtis

**Affiliations:** a Centre for Host-Microbiome Interactions, Faculty of Dentistry, Oral and Craniofacial Sciences, King’s College London, London, UK; b Department of Biomedical Sciences, College of Dentistry, King Faisal University, Saudi Arabia; c Department of Zoology, University of Oxford, Oxford, UK; d Institute of Evolutionary Biology, School of Biological Sciences, University of Edinburgh, Edinburgh, UK; e Department of Microbiology, Forsyth Institute, Cambridge, Massachusetts, USA; University of Trento

**Keywords:** 16S rRNA, *Streptococcus danieliae*, *Apodemus sylvaticus*, database, mouse models, *Mus musculus*, oral microbiology, oral microbiome

## Abstract

A curated murine oral microbiome database to be used as a reference for mouse-based studies has been constructed using a combination of bacterial culture, 16S rRNA gene amplicon, and whole-genome sequencing. The database comprises a collection of nearly full-length 16S rRNA gene sequences from cultured isolates and draft genomes from representative taxa collected from a range of sources, including specific-pathogen-free laboratory mice, wild Mus musculus *domesticus* mice, and formerly wild wood mouse *Apodemus sylvaticus*. At present, it comprises 103 mouse oral taxa (MOT) spanning four phyla—*Firmicutes*, *Proteobacteria*, *Actinobacteria*, and *Bacteroidetes*—including 12 novel undescribed species-level taxa. The key observations from this study are (i) the low diversity and predominantly culturable nature of the laboratory mouse oral microbiome and (ii) the identification of three major murine-specific oral bacterial lineages, namely, Streptococcus danieliae (MOT10), Lactobacillus murinus (MOT93), and *Gemella* species 2 (MOT43), which is one of the novel, still-unnamed taxa. Of these, *S. danieliae* is of particular interest, since it is a major component of the oral microbiome from all strains of healthy and periodontally diseased laboratory mice, as well as being present in wild mice. It is expected that this well-characterized database should be a useful resource for *in vitro* experimentation and mouse model studies in the field of oral microbiology.

**IMPORTANCE** Mouse model studies are frequently used in oral microbiome research, particularly to investigate diseases such as periodontitis and caries, as well as other related systemic diseases. We have reported here the details of the development of a curated reference database to characterize the oral microbial community in laboratory and some wild mice. The genomic information and findings reported here can help improve the outcomes and accuracy of host-microbe experimental studies that use murine models to understand health and disease. Work is also under way to make the reference data sets publicly available on a web server to enable easy access and downloading for researchers across the world.

## Abstract (precis)

Mouse model studies are frequently used in oral microbiome research, particularly to investigate diseases such as periodontitis and caries, as well as other related systemic diseases. We have reported here the details of the development of a curated reference database to characterize the oral microbial community in laboratory and some wild mice.

## INTRODUCTION

Mouse models play a crucial role in microbiome research, particularly in the investigation of the interactions between the host and the resident microbiota in health and disease (1, 2). Oral microbial population characterization in wild-type and mutant laboratory mouse strains have ranged from historical studies that involved identification of cultured isolates based on phenotypic characterization (3–6) to more recent molecular based studies focused on 16S rRNA gene sequencing (2, 7, 8). These studies have all demonstrated the mouse bacterial community to have a simple and relatively stable composition, with a major proportion of cultivable components, particularly in the specific-pathogen-free (SPF) laboratory mouse strains.

Despite this inherent simplicity of the mouse oral microbiome, it has significant relevance as a model to investigate and understand the mechanisms of human oral diseases such as periodontitis (7, 9, 10). A primary reason for this has been the parallels observed in the nature of the initiation and development of disease in experimental studies, specifically the development of a dysbiotic microbiome (characterized by increased total microbial loads) and soft tissue destruction with gingival inflammation (2, 11, 12), which are often comparable to that seen among humans (13, 14). Also, since the microbial genera observed are often similar to the predominant ones seen in humans (15), these animal models are also useful for understanding host-microbiota interactions and homeostasis mechanisms in health and disease.

However, the lack of adequate information in the public domain about mouse oral bacterial isolates from various sources, as well as poorly curated 16S rRNA gene sequences in the public databases, may lead to non- or misidentification of the organisms, which could thereby affect the outcome of such studies. A host-specific curated reference database for murine oral microbial populations in the public domain would enable researchers to accurately, reliably, and consistently identify the bacterial communities in experimental samples. Databases provide an improved and more accurate characterization of bacterial communities and allow easy comparison of work from different laboratories.

Similar databases have already been developed to characterize the oral microbiome in humans (15, 16) and other mammalian host species such as cats and dogs (17, 18). More recently, researchers have characterized and generated a database for the mouse intestinal bacterial community (19, 20), as well as chronicled the collection of genes in the mouse gut metagenome (21).

Here, we report a curated and well-characterized database of the oral bacterial population in mice, with representative genome sequences, which should greatly benefit researchers as a reference for oral microbiome studies in health and disease using laboratory mouse models.

## RESULTS

### Assignment of mouse oral taxa.

**FIG 1:**
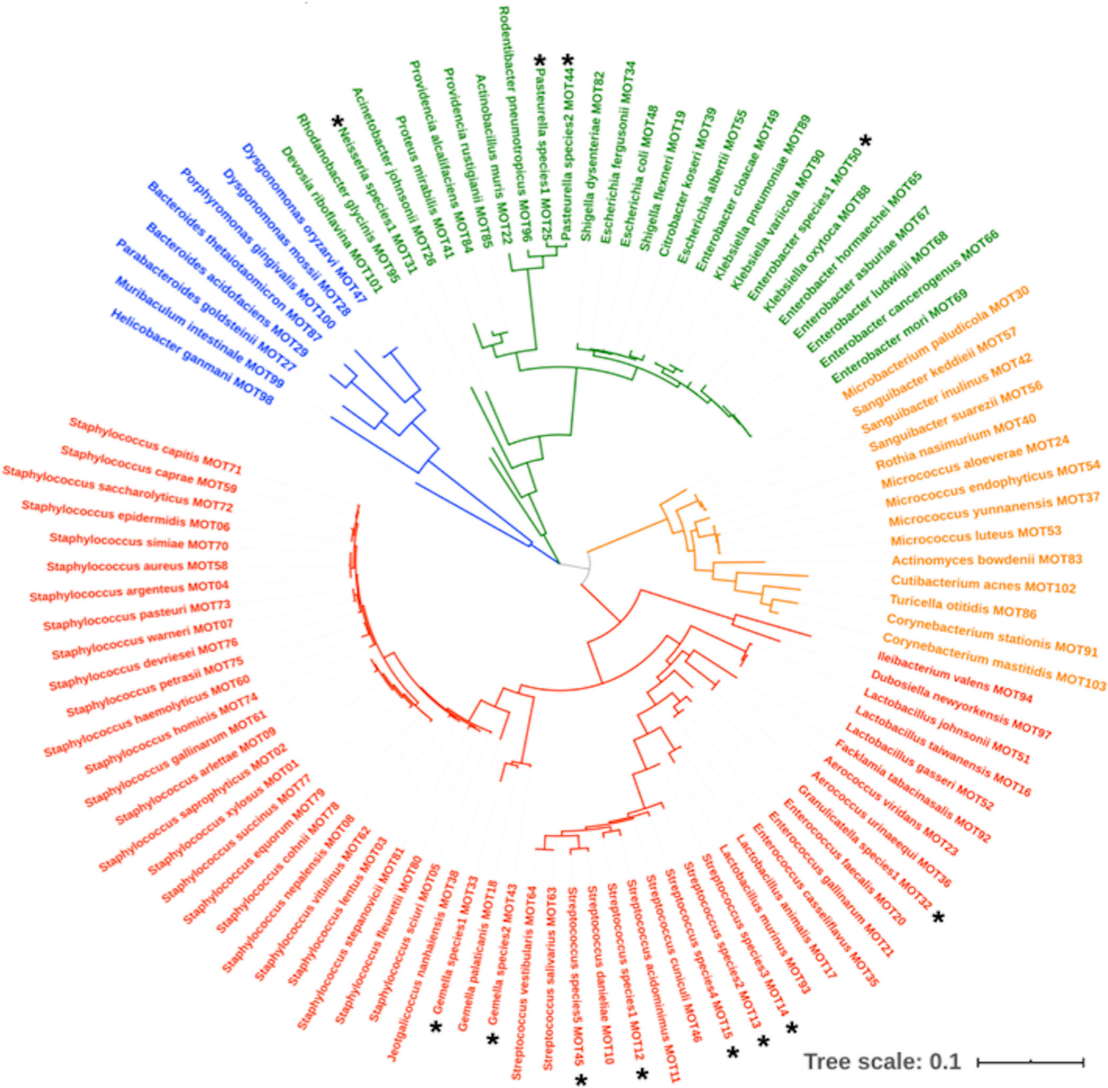
Taxonomic diversity in the murine oral microbiome database. The maximum-likelihood tree describes the phylogenetic relationships between the 103 mouse oral taxa (MOTs) identified in the murine oral microbiome database. Black asterisks indicate the MOTs of novel, previously unidentified species. Phylum clusters are color coded: *Firmicutes* (red), *Proteobacteria* (green), *Bacteroidetes* (blue), and *Actinobacteria* (orange).

To date, 325 16S rRNA gene sequences from murine oral bacterial isolates have been analyzed and found to constitute 103 mouse oral taxa (MOT) (Table 1 and Fig. 1). Twelve of the assigned MOTs (12.36%) represent novel, previously unidentified species that need further characterization in order to be assigned a formal species name. Representative 16S rRNA gene sequences for these novel taxa have been submitted to the National Center for Biotechnology Information (NCBI) database and are available under accession numbers MN095260 to MN095271. The 16S rRNA gene sequences for all the isolates analyzed in this study are available to download under accession numbers MW175535 to MW175859.

**TABLE 1:** Details of bacterial species and MOTs included in the murine oral microbiome database^*a*^

Taxonomy ID	Species ID	Type strain	16S rRNA gene accession no.	Source host
MOT01	Staphylococcus xylosus	ATCC 29971	D83374	Mus musculus */Apodemus sylvaticus*
MOT02	Staphylococcus saprophyticus	ATCC 15305	NR_115607	Mus musculus */Apodemus sylvaticus*
MOT03	Staphylococcus lentus	ATCC 29070	D83370	Mus musculus */Apodemus sylvaticus*
MOT04	Staphylococcus argenteus	MSHR1132	FR821777	Mus musculus
MOT05	Staphylococcus sciuri	DSM 20345T	AJ421446	Mus musculus */Apodemus sylvaticus*
MOT06	Staphylococcus epidermidis	ATCC 14990	D83363	Mus musculus
MOT07	Staphylococcus warneri	ATCC 27836	L37603	Mus musculus
MOT08	Staphylococcus nepalensis	DSM 15150	AJ517414	Mus musculus */Apodemus sylvaticus*
MOT09	Staphylococcus arlettae	ATCC 43957	AB009933	Mus musculus */Apodemus sylvaticus*
MOT10	Streptococcus danieliae	DSM 22233	GQ456229	Mus musculus
MOT11	Streptococcus acidominimus	LMG 17755	JX986969	Mus musculus
MOT12	*Streptococcus* species 1		MN095260	Mus musculus
MOT13	*Streptococcus* species 2		MN095261	Mus musculus
MOT14	*Streptococcus* species 3		MN095262	*Apodemus sylvaticus*
MOT15	*Streptococcus* species 4		MN095263	Mus musculus
MOT16	Lactobacillus taiwanensis	BCRC 17755	EU487512	Mus musculus
MOT17	Lactobacillus animalis	NBRC 15882	AB326350	Mus musculus */Apodemus sylvaticus*
MOT18	Gemella palaticanis	CCUG 39489	Y17280	Mus musculus
MOT19	Shigella flexneri	ATCC 29903	X96963	Mus musculus
MOT20	Enterococcus faecalis	JCM 5803	AB012212	Mus musculus
MOT21	Enterococcus gallinarum	ATCC 49573	AF039900	Mus musculus
MOT22	Muribacter muris	NCTC 12432	AY362894	Mus musculus
MOT23	Aerococcus viridans	NCTC 8251	AB680262	Mus musculus
MOT24	Micrococcus aloeverae	DSM 27472	KF524364	Mus musculus
MOT25	*Pasteurella* species 1		MN095264	*Apodemus sylvaticus*
MOT26	Acinetobacter johnsonii	ATCC 17909	Z93440	Mus musculus
MOT27	Parabacteroides goldsteinii	WAL 12034	AY974070	Mus musculus
MOT28	Dysgonomonas mossii	CCUG 43457	AJ319867	Mus musculus
MOT29	Bacteroides acidifaciens	JCM 10556	AB021164	Mus musculus
MOT30	Microbacterium paludicola	DSM 16915	AJ853909	Mus musculus
MOT31	*Neisseria* species 1		MN095265	*Apodemus sylvaticus*
MOT32	*Granulicatella* species 1		MN095266	*Apodemus sylvaticus*
MOT33	*Gemella* species 1		MN095267	*Apodemus sylvaticus*
MOT34	Escherichia fergusonii	ATCC 35469	AF530475	Mus musculus
MOT35	Enterococcus casseliflavus	ATCC 700327	AF039903	Mus musculus
MOT36	Aerococcus urinaeequi	ATCC 29723	D87677	Mus musculus
MOT37	Micrococcus yunnanensis	YIM 65004	FJ214355	Mus musculus
MOT38	Jeotgalicoccus nanhaiensis	JSM 077023	FJ237390	Mus musculus
MOT39	Citrobacter koseri	ATCC 27028	HQ992945	Mus musculus
MOT40	Rothia nasimurium	CCUG 35957	AJ131121	Mus musculus
MOT41	Proteus mirabilis	ATCC 29906	DQ885256	Mus musculus
MOT42	Sanguibacter inulinus	NCFB 3024	X79451	Mus musculus
MOT43	*Gemella* species 2		MN095268	Mus musculus
MOT44	*Pasteurella* species 2		MN095269	*Apodemus sylvaticus*
MOT45	*Streptococcus* species 5		MN095270	*Apodemus sylvaticus*
MOT46	Streptococcus cuniculi	CCUG 65085	HG793791	*Apodemus sylvaticus*
MOT47	Dysgonomonas oryzarvi	JCM 16859	AB547446	Mus musculus
MOT48	Escherichia coli	ATCC 11775	X80725	Mus musculus
MOT49	Enterobacter cloacae	ATCC 13047	AJ251469	Mus musculus
MOT50	*Enterobacter* species 1		MN095271	Mus musculus
MOT51	Lactobacillus johnsonii	ATCC 33200	AJ002515	Mus musculus
MOT52	Lactobacillus gasseri	ATCC 33323	AF519171	Mus musculus
MOT53	Micrococcus luteus	DSM 20030	AJ536198	Mus musculus
MOT54	Micrococcus endophyticus	DSM 17945	EU005372	Mus musculus
MOT55	Escherichia albertii	CCUG 46494	AJ508775	Mus musculus
MOT56	Sanguibacter suarezii	ATCC 51766	X79452	Mus musculus
MOT57	Sanguibacter keddieii	ATCC 51767	X79450	Mus musculus
MOT58	Staphylococcus aureus	ATCC 12600	L36472	Mus musculus
MOT59	Staphylococcus caprae	ATCC 35538	AB009935	Mus musculus
MOT60	Staphylococcus haemolyticus	ATCC 29970	X66100	Mus musculus
MOT61	Staphylococcus gallinarum	ATCC 35539	D83366	Mus musculus */Apodemus sylvaticus*
MOT62	Staphylococcus vitulinus	ATCC 51145	AB009946	Mus musculus */Apodemus sylvaticus*
MOT63	Streptococcus salivarius	ATCC 7073	AY188352	Mus musculus
MOT64	Streptococcus vestibularis	NCTC 12167	AY188353	Mus musculus
MOT65	Enterobacter hormaechei	ATCC 49162	AJ508302	Mus musculus
MOT66	Enterobacter cancerogenus	ATCC 33241	Z96078	Mus musculus
MOT67	Enterobacter asburiae	ATCC 35953	AB004744	Mus musculus
MOT68	Enterobacter ludwigii	CCUG 51323	AJ853891	Mus musculus
MOT69	Enterobacter mori	LMG 25706	EU721605	Mus musculus
MOT70	Staphylococcus simiae	LMG 22723	AY727530	Mus musculus
MOT71	Staphylococcus capitis	ATCC 27840	L37599	Mus musculus
MOT72	Staphylococcus saccharolyticus	ATCC 14953	L37602	Mus musculus
MOT73	Staphylococcus pasteuri	ATCC 51129	AB009944	Mus musculus
MOT74	Staphylococcus hominis	ATCC 27844	X66101	Mus musculus
MOT75	Staphylococcus petrasii	CCUG 62727	JX139845	Mus musculus
MOT76	Staphylococcus devriesei	CCUG 58238	FJ389206	Mus musculus
MOT77	Staphylococcus succinus	ATCC 700337	AF004220	Mus musculus */Apodemus sylvaticus*
MOT78	Staphylococcus cohnii	ATCC 29974	D83361	Mus musculus */Apodemus sylvaticus*
MOT79	Staphylococcus equorum	ATCC 43958	AB009939	Mus musculus */Apodemus sylvaticus*
MOT80	*Staphylococcus fleuretti*	ATCC BAA-274	AB233330	Mus musculus */Apodemus sylvaticus*
MOT81	Staphylococcus stepanovicii	CCM 7717	GQ222244	Mus musculus */Apodemus sylvaticus*
MOT82	Shigella dysenteriae	ATCC 13313	X96966	Mus musculus
MOT83	Actinomyces bowdenii	DSM 15435	AJ234039	Wild Mus musculus
MOT84	Providencia alcalifaciens	ATCC 9886T	AJ301684	Wild Mus musculus
MOT85	Providencia rustigianii	DSM 4541	AM040489	Wild Mus musculus
MOT86	Turicella otitidis	DSM 8821	X73976	Mus musculus
MOT87	*Bacteroides thetaiotaomicron*	NCTC 10582	NR_074277	Mus musculus
MOT88	Klebsiella oxytoca	ATCC 13182	AF129440	Mus musculus
MOT89	Klebsiella pneumoniae	ATCC 13883	X87276	Mus musculus
MOT90	*Klebsiella variicola*	DSM 15968	AJ783916	Mus musculus
MOT91	*Corynebacterium stationis*	DSM 20302	FJ172667	Mus musculus
MOT92	*Facklamia tabacinasalis*	ATCC 700838	Y17820	Mus musculus
MOT93	Lactobacillus murinus	ATCC 35020	AJ621554	Mus musculus
MOT94	Ileibacterium valens	DSM 103668T	NR_156909	Mus musculus
MOT95	Rhodanobacter glycinis	NBRC 105007	EU912469	Mus musculus
MOT96	*Rodentibacter pneumotropicus*	ATCC 35149	M75083	Mus musculus
MOT97	Dubosiella newyorkensis	DSM 103457T	NR_156910	Mus musculus
MOT98	Helicobacter ganmani	CCUG 43526	AF000221	Mus musculus
MOT99	*Muribaculum intestinale*	DSM 28989	KR364784	Mus musculus
MOT100	Porphyromonas gingivalis	ATCC 33277	AF414809	Mus musculus
MOT101	Devosia riboflavina	ATCC 9526	AJ549086	Mus musculus
MOT102	*Cutibacterium acnes*	ATCC 6919	X53218	Mus musculus
MOT103	*Corynebacterium mastitidis*	DSM 44356	Y09806	Wild Mus musculus

a Accession numbers are provided for 16S rRNA gene sequences of the type strains of named species.

### Diversity of the murine oral bacterial community.

The mouse oral taxa are distributed across four bacterial phyla (Fig. 1): *Firmicutes* (54 taxa), *Proteobacteria* (27 taxa), *Actinobacteria* (14 taxa), and *Bacteroidetes* (8 taxa). The *Firmicutes* phylum, having the greatest number of taxa, is represented by species of the *Streptococcus*, *Staphylococcus*, *Lactobacillus*, *Gemella*, *Enterococcus*, *Aerococcus*, *Jeotgalicoccus*, *Granulicatella*, *Facklamia*, *Dubosiella*, and *Ileibacterium* genera. *Proteobacteria* are comprised of members of the *Enterobacter*, *Klebsiella*, *Shigella*, *Escherichia*, *Pasteurella*, *Providencia*, *Proteus*, *Actinobacillus*, Acinetobacter, *Neisseria*, *Rodentibacter*, *Rhodanobacter*, and *Devosia* genera. The *Bacteroidetes* phylum comprises the *Bacteroides*, *Parabacteroides*, *Porphyromonas*, *Helicobacter*, *Muribaculum*, and *Dysgonomonas* genera, while *Actinobacteria* are represented by the *Micrococcus*, *Sanguibacter*, *Microbacterium*, *Rothia*, *Corynebacterium*, *Actinomyces*, *Cutibacterium*, and *Turicella* genera. At the species level, the species Streptococcus danieliae MOT10 was most frequently observed among the samples tested. The periodontal pathogen Porphyromonas gingivalis, albeit never isolated in the laboratory from the murine swab samples, was identified among the amplicon sequencing data of the 16S rRNA gene. The species has been observed in the wild mouse species and occasionally in experimental SPF laboratory mice, but never in healthy, untreated SPF mice.

### Laboratory versus wild mouse oral bacterial community.

**FIG 2:**
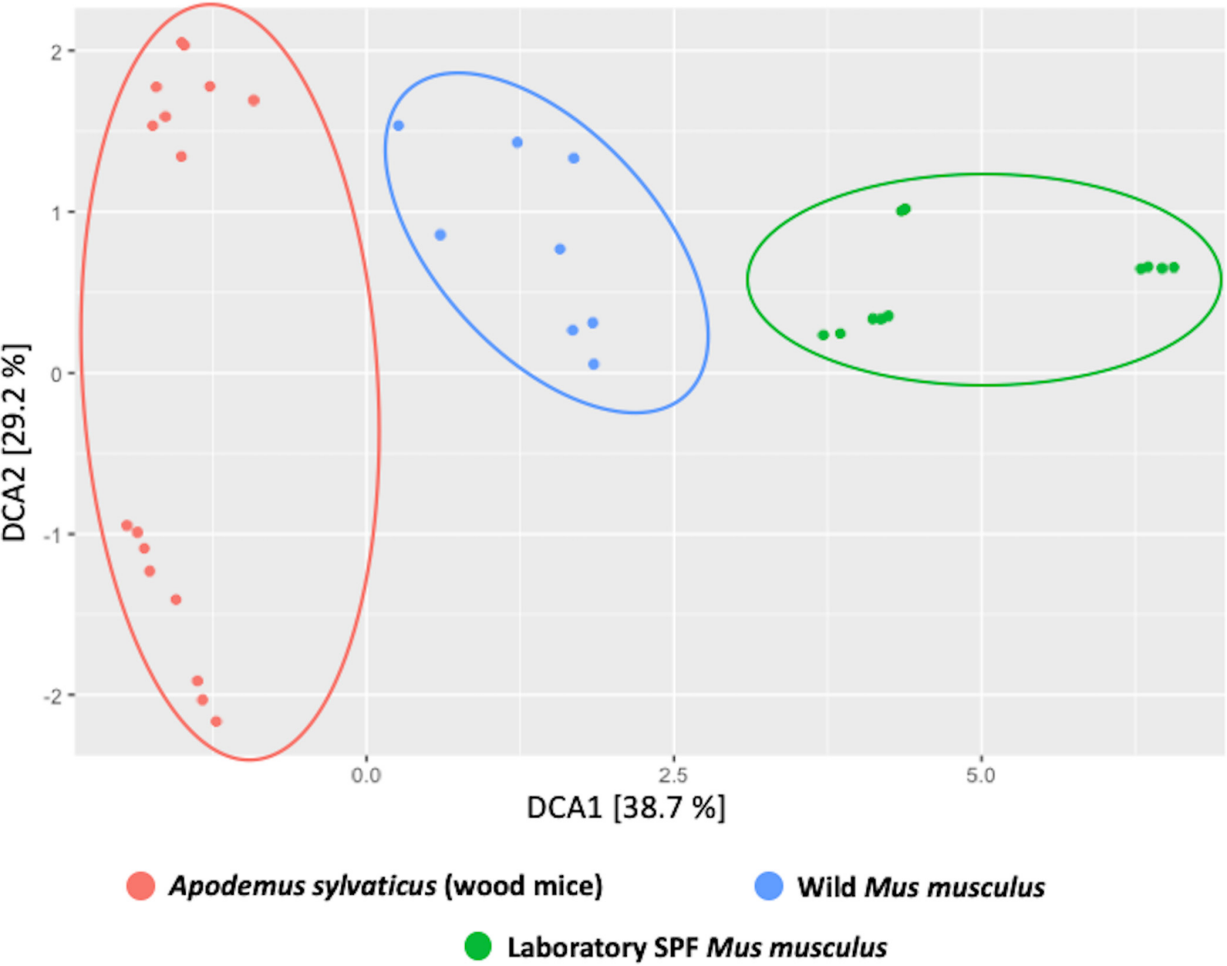
Beta-diversity analyses of the oral microbiome of various mouse species. Beta-diversity analyses of oral microbial populations of SPF laboratory mice (green), wild Mus musculus *domesticus* mice (blue), and wood mice *Apodemus sylvaticus* (red) using the 16S rRNA gene amplicon community data targeting the V1-V2 region were performed.

The SPF laboratory mice included in this study represented diverse background strains (C57BL/6J, C3H/Orl, CD-1, BALB/c) and were also obtained from a range of diverse sources (commercially purchased, in-house colonies, conventionalized from germfree mice, and genetic knockouts). In addition, samples were also collected from the formerly wild wood mouse *Apodemus sylvaticus* and the wild house mouse Mus musculus *domesticus.* Beta-diversity analyses of the oral microbial populations of the sampled mice (Fig. 2) showed distinct separation in the clustering of all three groups of mice. However, a number of shared species have also been observed between them, including Lactobacillus murinus (MOT93) and Streptococcus danieliae (MOT10) (Table 1).

### Culture versus 16S rRNA gene community profiling.

**FIG 3:**
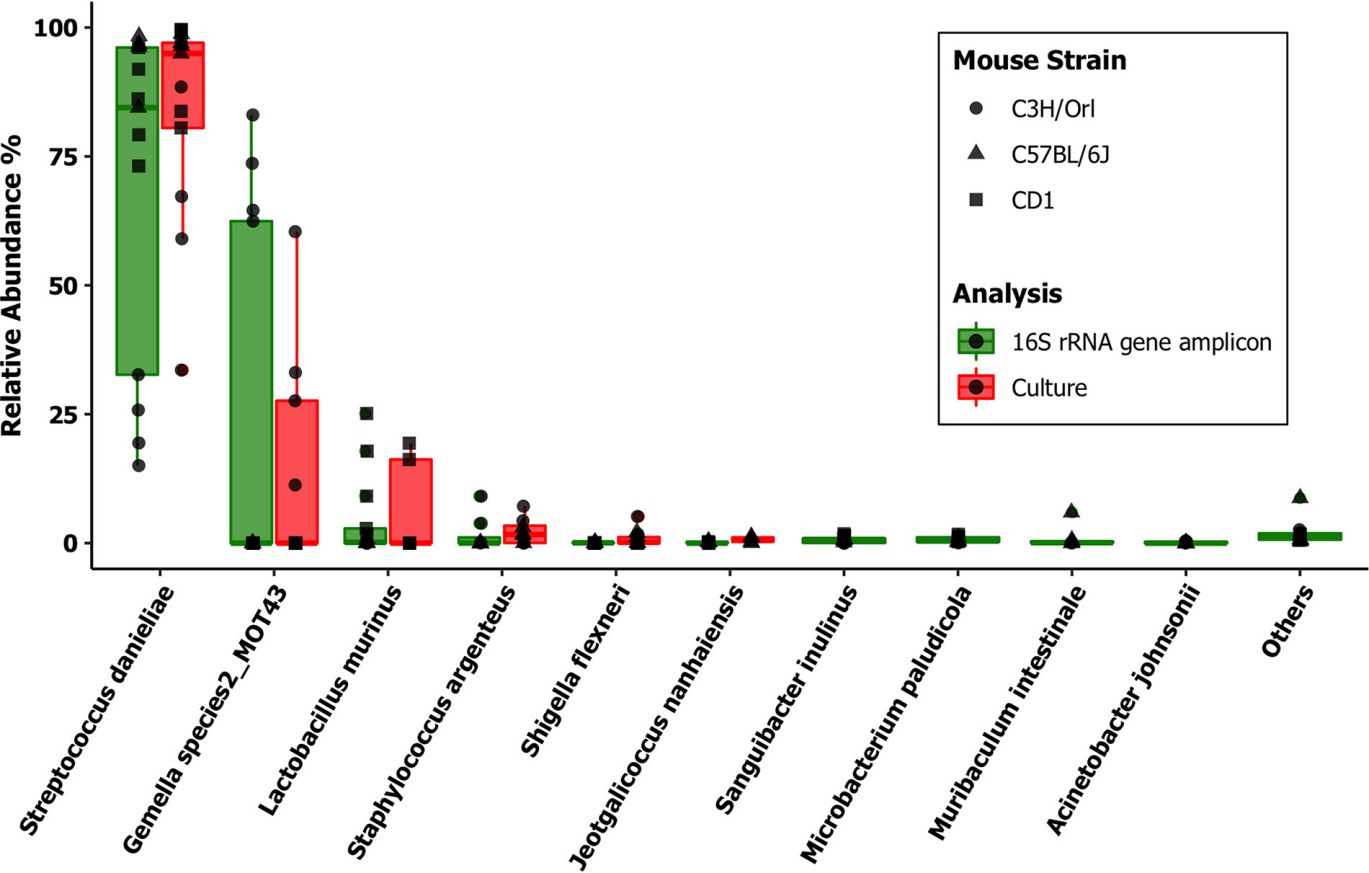
Relative abundances in the SPF laboratory mice (*n* = 13). We compared oral microbial population analyses in three background strains of SPF mice by laboratory culture (red) and Illumina MiSeq 16S rRNA gene amplicon sequencing (green) amplifying the V1-V2 region of the 16S rRNA gene. The amplicon sequencing data were analyzed using DADA2 v1.8 against a custom-made taxonomy reference data set from this database. The top 10 most relatively abundant taxa identified after normalization of counts are listed.

**FIG 4:**
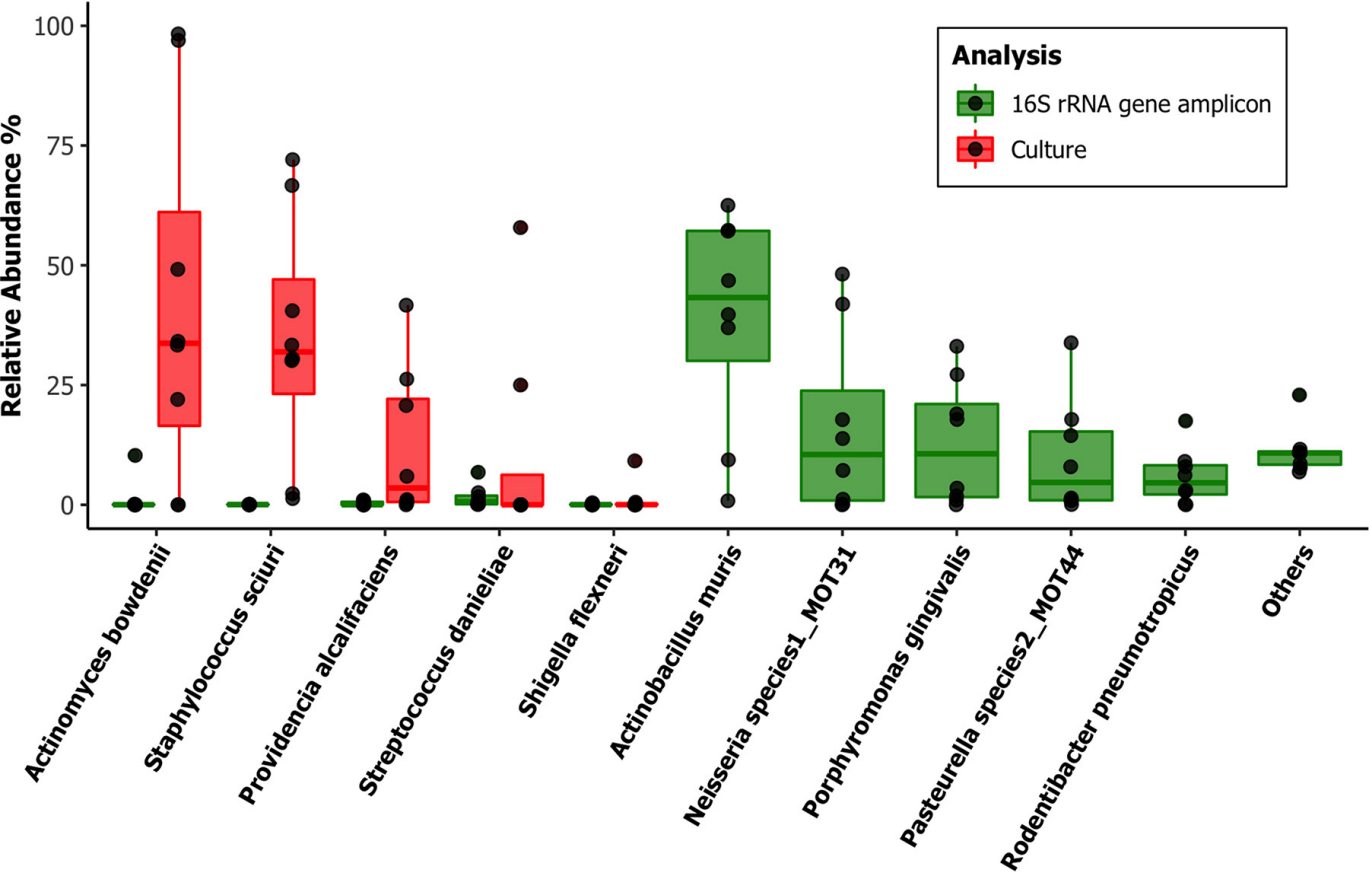
Relative abundances in the wild house mouse Mus musculus *domesticus* (*n* = 8). We compared oral microbial population analyses in the wild house mouse Mus musculus *domesticus* by laboratory culture (red) and Illumina MiSeq 16S rRNA gene amplicon sequencing (green) amplifying the V1-V2 region of the 16S rRNA gene. The amplicon sequencing data were analyzed using DADA2 v1.8 against a custom-made taxonomy reference data set from this database. The top 10 most relatively abundant taxa identified after normalization of counts are listed.

Custom reference data sets for community profiling analysis were constructed using the current version of the database and used for data analysis. Analysis of the Illumina MiSeq amplicon sequencing data (the V1-V2 region of the 16S rRNA gene) in SPF mouse samples using the custom reference data set revealed that 98% of the total number of reads could be assigned to species level. Further comparative analysis of culturing data with this amplicon sequencing data set revealed a significant consensus in the SPF mouse samples, with all of the cultured species being represented at comparable levels with the next-generation sequencing (NGS) data (Fig. 3). Among the wild Mus musculus *domesticus* samples, 95% of the reads were identified to the species level using the custom reference data set, even though very little consensus has been observed with the culturing data in terms of abundance of individual species (Fig. 4). This could be explained by the fact that the species that show significantly higher abundance in the NGS data set (Muribacter muris, *Neisseria* species 1, and Porphyromonas gingivalis) are predominantly slow growers and therefore could have been missed being detected in the 48-h culturing protocol followed in this study.

**FIG 5:**
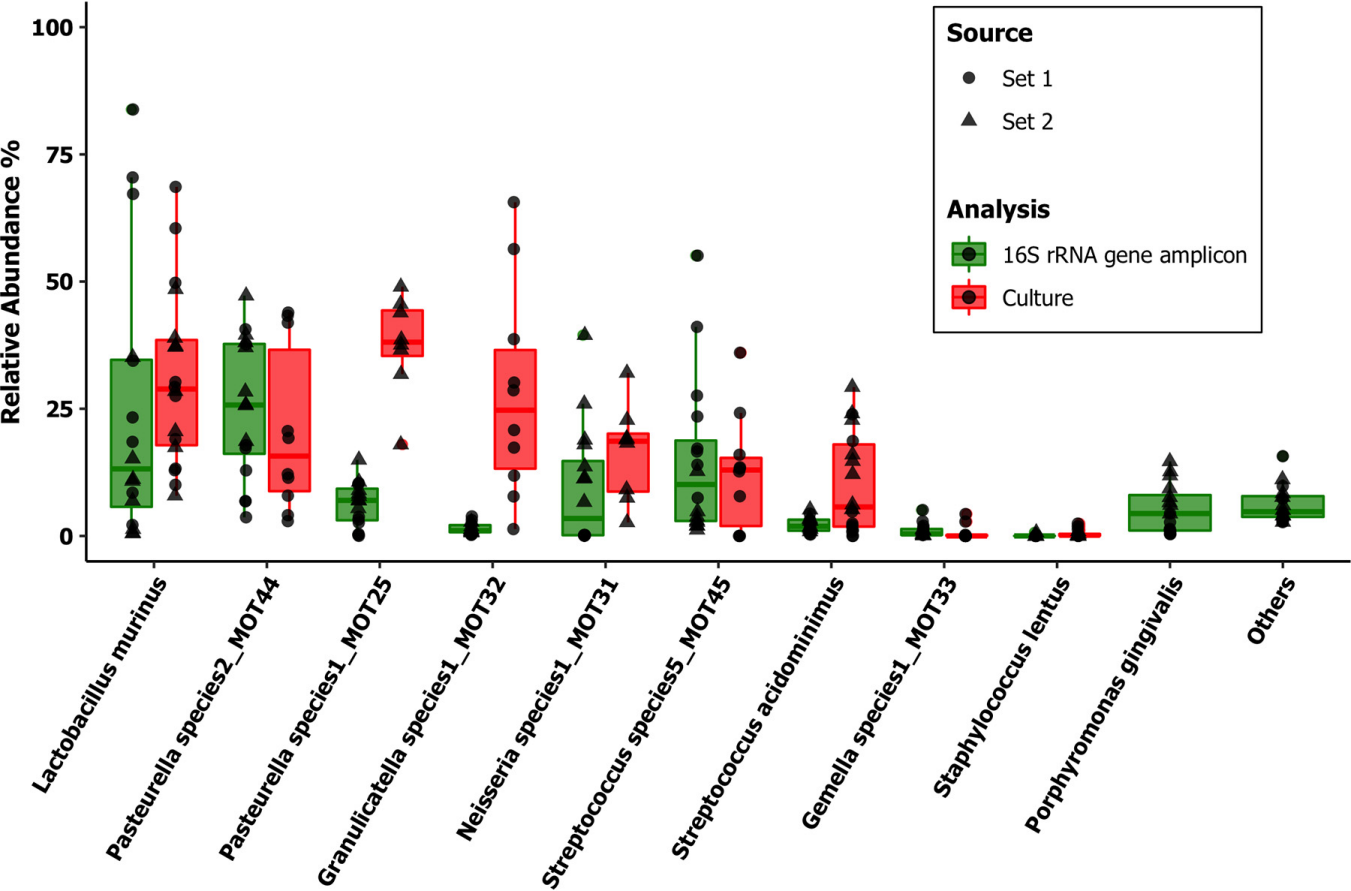
Relative abundances in the wood mouse *Apodemus sylvaticus* (*n* = 16). We compared oral microbial population analyses in the formerly wild wood mouse *Apodemus sylvaticus*, sampled from two sources (sets 1 and 2), by laboratory culture (red) and Illumina MiSeq 16S rRNA gene amplicon sequencing (green) amplifying the V1-V2 region of the 16S rRNA gene. The amplicon sequencing data were analyzed using DADA2 v1.8 against a custom-made taxonomy reference data set from this database. The top 10 most relatively abundant taxa identified after normalization of counts are listed.

In the samples from the formerly wild wood mouse *Apodemus sylvaticus*, the proportion of reads positively identified using the custom data set fell to 83%, even though a higher consensus between culturing and NGS was observed compared to the wild Mus musculus *domesticus*, despite the increased diversity (Fig. 5).

Current versions of the reference data sets have been constructed for both the mothur and DADA2 pipelines and are available to download at https://figshare.com/s/2470f05ab77cdf40b2f8.

### Mouse oral bacterial genomes.

Draft genomes of 55 murine oral bacterial isolates have been generated, sourced from all the mouse groups included in this study (Table 2). Representative genome sequences have been uploaded to the NCBI bacterial genome database and are publicly available at NCBI BioProject PRJNA671681. After assembly, the genomes had a mean contig number of 135 (±134) and GC% ratios ranging from 28 to 73%.

**TABLE 2:** Details of the draft microbial genomes sequenced as part of the development of the murine oral microbiome database^*a*^

Sample ID	Species ID	Taxonomy ID	Total length (bp)	GC%	Source	Accession no.
19428wA1_WT6	Staphylococcus xylosus	MOT01	2806698	32.68	SPF C3H/Orl	JADGLO000000000
19428wB1_WM06	Staphylococcus saprophyticus	MOT02	2920674	32.48	*Apodemus sylvaticus*	JADGLJ000000000
3_3BalbC2O2_S3	Staphylococcus lentus	MOT03	2925394	32.36	SPF BALB/C	JADGLT000000000
19428wC1_HT5	Staphylococcus lentus	MOT03	2910603	31.73	SPF C3H/Orl	JADGLE000000000
19428wG1_AE2	Staphylococcus lentus	MOT03	2856564	31.81	SPF C57BL/6J	JADGMD000000000
ACT.1	Staphylococcus sciuri	MOT05	2737620	32.5	SPF C3H/Orl	JADGMB000000000
19428wE1_BL01	Staphylococcus sciuri	MOT05	2833136	32.42	SPF C57BL/6J	JADGMG000000000
19428wF1_P912	Staphylococcus warneri	MOT07	2628448	32.57	SPF C3H/Orl	JADGMF000000000
19428wH1_IOV5	Staphylococcus arlettae	MOT09	2600982	33.35	SPF C3H/Orl	JADGMC000000000
1_1BalbC1O2_S1	Streptococcus danieliae	MOT10	1356823	44.6	SPF BALB/c	JADGLV000000000
STR.1	Streptococcus danieliae	MOT10	1969143	44.5	SPF C3H/Orl	JADGKG000000000
CCW311.1	Streptococcus danieliae	MOT10	1616067	45.03	SPF C57BL/6J	JADGKO000000000
5_7CXCR22O2_S5	Streptococcus danieliae	MOT10	2927540	43.29	SPF BALB/c	JADGLY000000000
19428wB2_HT4	Streptococcus acidominimus	MOT11	2221121	42.77	SPF C3H/Orl	JADGLI000000000
6_8CXCR23O2_S6	Streptococcus acidominimus	MOT11	2509022	43.7	SPF BALB/c	JADGLR000000000
19428wC2_LYSM12	*Streptococcus* species 1	MOT12	2417981	40.73	SPF C3H/Orl	JADGLD000000000
19428wD3_AN2	*Streptococcus* species 2	MOT13	2324918	42.8	SPF C3H/Orl	JADGKY000000000
CIP106318T.1	Gemella palaticanis	MOT18	1720464	27.55	Culture collection, type strain	JADGKN000000000
19428wH2_1568	Shigella flexneri	MOT19	5100000	50.71	SPF C3H/Orl	JADGKR000000000
19428wA3_CC11	Enterococcus faecalis	MOT20	6434324	39.02	SPF C3H/Orl	JADGLM000000000
19428wB3_C2	Enterococcus gallinarum	MOT21	3661725	40.12	SPF C3H/Orl	JADGLH000000000
ENT.1	Enterococcus gallinarum	MOT21	3793521	40	SPF C3H/Orl	JADGKL000000000
19428wC3_WT12	Muribacter muris	MOT22	2520282	44.93	SPF C3H/Orl	JADGLC000000000
7_18CXCR221ANO_S7	Muribacter muris	MOT22	2535311	45.96	SPF BALB/c	JADGLQ000000000
19428wE3_DK2	Micrococcus aloeverae	MOT24	2559064	72.93	SPF C57BL/6J	JADGLX000000000
19428wF3_WM03	*Pasteurella* species 1	MOT25	2264212	39.91	*Apodemus sylvaticus*	JADGKV000000000
19428wG3_P14	Parabacteroides goldsteinii	MOT27	6779962	43.44	SPF C3H/Orl	JADGKT000000000
19428wE4_P11	Dysgonomonas mossii	MOT28	4110280	37.31	SPF C3H/Orl	JADGKW000000000
8_33DSS_C5_S8	Bacteroides acidifaciens	MOT29	5228201	44.3	SPF C3H/Orl	JADGLP000000000
19428wH3_P2318	Bacteroides acidifaciens	MOT29	5116387	43.26	SPF C3H/Orl	JADGKQ000000000
19428wA4_P1012	Microbacterium paludicola	MOT30	2809652	70.53	SPF C3H/Orl	JADGLL000000000
19428wB4_WF04	*Neisseria* species 1	MOT31	2568531	53.59	*Apodemus sylvaticus*	JADGLG000000000
19428wC4_WM01	*Granulicatella* species 1	MOT32	2003757	33.14	*Apodemus sylvaticus*	JADGLB000000000
19428wG4_IOV6	Micrococcus yunnanensis	MOT37	2475752	73.07	SPF C3H/Orl	JADGKS000000000
19428wE5_W307	Jeotgalicoccus nanhaiensis	MOT38	2244448	41.43	SPF C57BL/6J	JADGLW000000000
19428wH4_BL23	Citrobacter koseri	MOT39	4821534	53.81	SPF C57BL/6J	JADGKP000000000
19428wA5_irhom_31	Rothia nasimurium	MOT40	2677034	59.02	SPF C57BL/6J	JADGLK000000000
19428wB5_irhom_Sw	Proteus mirabilis	MOT41	3939005	38.79	SPF C57BL/6J	JADGLF000000000
DSM100099.1	Sanguibacter inulinus	MOT42	4257809	70.83	Culture Collection; Type Strain	JADGKM000000000
19428wG2_WT2a	*Gemella* species 2	MOT43	1404407	30.33	SPF C3H/Orl	JADGKU000000000
GH3.1	*Gemella* species 2	MOT43	1563132	28.2	SPF C3H/Orl	JADGKK000000000
GL1.1	*Gemella* species 2	MOT43	1299399	30.25	SPF C3H/Orl	JADGKJ000000000
19428wA2_WM07	*Streptococcus* species 5	MOT45	1714730	41.26	*Apodemus sylvaticus*	JADGLN000000000
19428wD2_WM131	Streptococcus cuniculi	MOT46	2407100	42.7	*Apodemus sylvaticus*	JADGKZ000000000
19428wD4_HM13	Escherichia coli	MOT48	5021976	50.62	SPF C3H/Orl	JADGMH000000000
19428wE2_CC3	Lactobacillus johnsonii	MOT51	1903680	34.48	SPF C3H/Orl	JADGKX000000000
4_5BalbC4O2_S4	Staphylococcus aureus	MOT58	2825768	33.69	SPF BALB/c	JADGLS000000000
19428wD1_P36	Staphylococcus aureus	MOT58	2692071	32.75	SPF C3H/Orl	JADGLA000000000
STF.1	Staphylococcus aureus	MOT58	2699934	32.8	SPF C3H/Orl	JADGKH000000000
GW2.1	Staphylococcus capitis	MOT71	2450948	32.8	SPF C3H/Orl	JADGMA000000000
WMus004.1	Actinomyces bowdenii	MOT83	3174558	71.17	Wild Mus musculus *domesticus*	JADGKF000000000
WMus003.1	Providencia alcalifaciens	MOT84	3909646	41.88	Wild Mus musculus *domesticus*	JADGLZ000000000
2_2BalbC1BO2_S2	Lactobacillus murinus	MOT93	2341418	40.54	SPF BALB/c	JADGLU000000000
19428wF2_HT12	Lactobacillus murinus	MOT93	2254694	39.68	SPF C3H/Orl	JADGME000000000
LAC.1	Lactobacillus murinus	MOT93	2089346	40.1	SPF C3H/Orl	JADGKI000000000

a The 55 draft genomes are available to download from BioProject (PRJNA671681), while the individual accession numbers have been provided in the table itself.

### Predominant bacterial genera in laboratory mice.

The following three genera were found to be of particular interest due to their presence in a wide range of samples tested.

**FIG 6:**
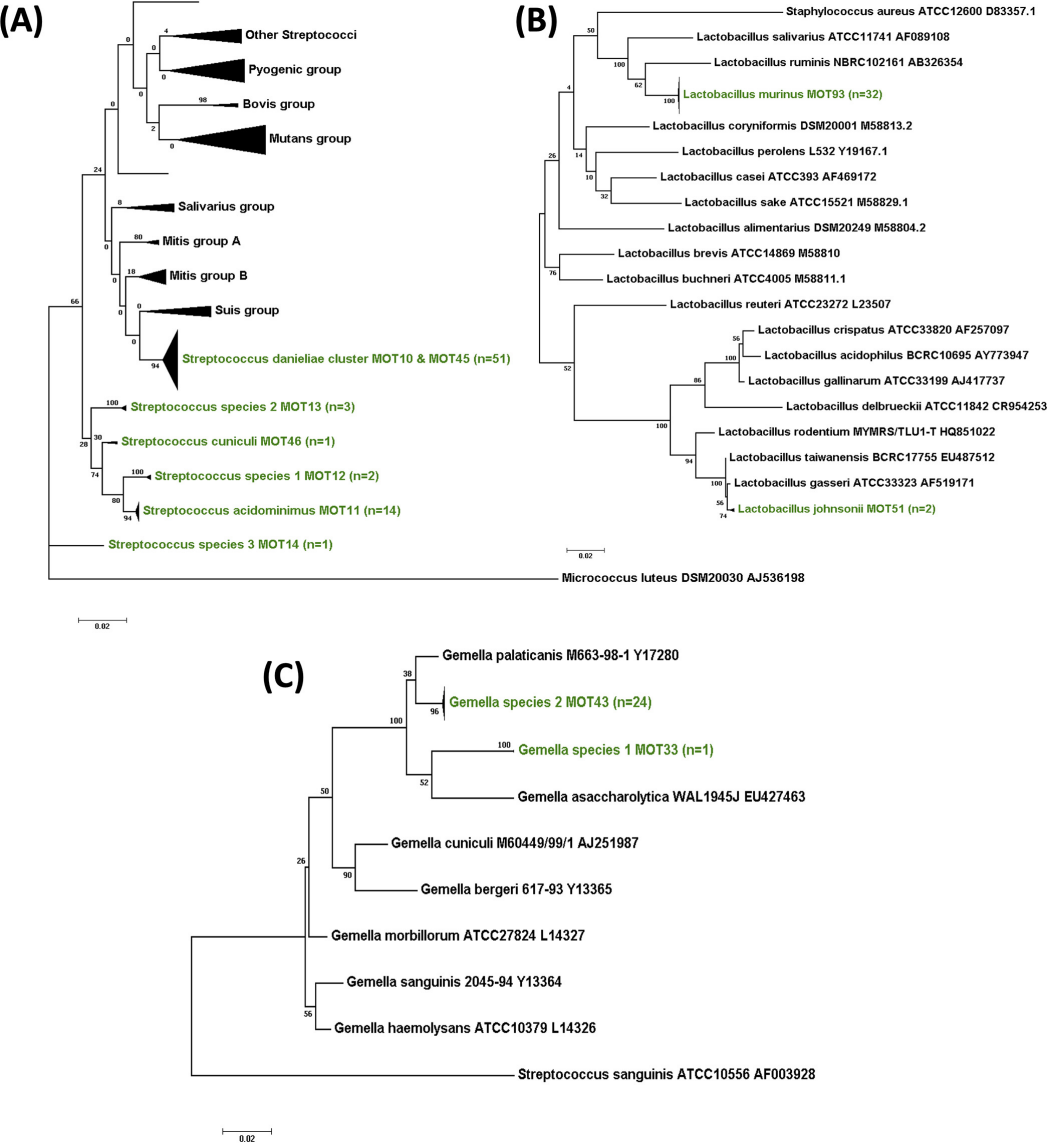
Phylogenetic diversity of the predominant bacterial genera in laboratory mice. Maximum-likelihood phylogenetic trees represent the diversity in murine oral bacterial isolates for the three most abundant genera: *Streptococcus* (A), *Lactobacillus* (B), and *Gemella* (C). Each tree has been rooted with an outlier and constructed using 100 bootstrap replicates. The branches in green refer to the taxonomic IDs in each genus of the murine isolates from this study.

**(i) *Streptococcus*.** *S. danieliae* MOT10 was the most commonly detected species in all SPF laboratory mouse groups from multiple sources and mouse strains (Fig. 6A). This species was also isolated from the wild Mus musculus *domesticus*. *Streptococcus* species 5 MOT45 was isolated from the wood mouse *A. sylvaticus* (Fig. 5) and was found to be very closely related to, but distinct from, *S. danieliae* (16S rRNA gene sequence similarity, 98.4%) forming a murine *S. danieliae* cluster.

Some isolates of *S. acidominimus* MOT11 were observed in a group of SPF mice belonging to the BALB/c strain, whereas a few novel streptococcal species were also isolated in single occurrences (*Streptococcus* species 1, 2, and 3; MOTs 12 to 14).

**(ii) *Lactobacillus*.** With only two exceptions, all of the lactobacilli in mice were identified as *L. murinus* MOT93 (Fig. 6B). The two exceptions belonged to the *L. taiwanensis-gasseri-johnsonii* cluster at >99% identity and could not be distinguished based on the 16S rRNA gene sequence analysis. *In silico* DNA-DNA hybridization gene sequence analysis identified them as L. johnsonii MOT51 at 72.1% genome identity.

**(iii) *Gemella*.** All *Gemella* isolates from laboratory mice were found to belong to a novel, as yet unnamed, species, *Gemella* species 2 MOT43 (Fig. 6C). The nearest phylogenetic neighbor is the canine oral species, Gemella palaticanis at 97.4% 16S rRNA gene sequence identity. Another single, novel *Gemella* isolate was observed in *A. sylvaticus* wood mice and designated *Gemella* species 1 MOT33.

## DISCUSSION

We report here the details of a curated murine database to represent the diversity of the oral microbiome in laboratory and wild mouse populations.

The low diversity of the mouse oral bacterial community, especially in SPF laboratory mice, has particularly stood out in this characterization. The presence of such a small and specifically targeted population makes accurate species level identification especially significant for improving the outcomes of studies. In addition, the collection of draft genomes of the representative MOTs from the database is also of great benefit for microbial population studies in mice. This repository of genomes should enable the generation of quality reference data sets for metagenomic and metatranscriptomic analyses of mouse-based oral studies.

A comparative analysis of the culture and 16S rRNA gene community profiling data has further confirmed the low diversity of the lab mouse oral microbiome (see Fig. S1 in the supplemental material), which is dominated by four species-level taxa. We have reported here only a representative subset of the samples we have sequenced in order to demonstrate the development of the database. However, over the course of multiple experimental studies, we have shown the consistency in the results of these population analyses (2, 7). The diversity of oral species-level taxa, in comparison, was found to be higher in the wild mouse species. Such variations in the microbial diversity and load within host species has been reported in the gut microbiome of other organisms and has largely been attributed to the influence of diet and environment (22, 23).

10.1128/mSystems.01222-20.5 FIG S1 Alpha-diversity analyses of the oral microbiome of various mouse species. Alpha-diversity analyses were carried out using Shannon and Simpson diversity indices comparing the oral microbial populations of SPF laboratory mice (green), wild Mus musculus *domesticus* (blue) mice, and formerly wild wood mice, *Apodemus sylvaticus* (red). Download FIG S1, TIF file, 2.5 MB. Copyright © 2021 Joseph et al. 2021 Joseph et al. This content is distributed under the terms of the Creative Commons Attribution 4.0 International license.

Another observation of this study has been the variations observed between different strains of the laboratory mice; we have previously also observed this between batches of the same strain of mice obtained from commercial sources (unpublished data), which has also been reported in some older studies (24). Recently, Abusleme et al. also reported the variations observed in the oral microbial communities in C57BL/6 mice purchased from two major commercial animal suppliers and the increased stability of a particular type of population when the mice were cohoused (8). This raises a very important issue of having a well-curated reference database, as well as internal controls, to ensure accuracy in results in microbiome studies.

Among the three predominant host-specific MOTs identified, *S. danieliae* MOT10 is of particular interest due to its dominance of the oral bacterial community in the SPF laboratory mice. A recently proposed species of the *Streptococcus* genus, *S. danieliae* MOT 10 was originally described based on a murine cecal isolate for which the authors suggested an oral/upper respiratory tract origin (25). There have been few other reports of the organism so far (26–28), all of them of murine origin. The organism has also been reported to be one of the key drivers in the establishment of the oral microbial community in laboratory SPF mice after the eruption of teeth (8). *Gemella* species 2 has been isolated from oral samples of multiple strains of laboratory mice and needs further characterization to be taxonomically identified as an official bacterial species. Of these three species, *L. murinus* is the only species that was part of the Altered Schaedler Formula, the community of microbes that were used to originally colonize gnotobiotic mice to develop SPF laboratory mice as we know them today (29, 30). Similar examples of host specificity have also been reported in other mouse microbiomes, including the colonization of segmented filamentous bacteria (31) and *Muribaculaceae* (19) in the mouse gut microbiome. This specificity also strongly indicates to the phenomenon of host-microbe coevolution and fitness characteristics (32).

In addition, there has been an increasing interest in recent times in the relevance of wild rodent models in research (33, 34), particularly for understanding natural progressions of certain diseases in relation to the microbiome. A study involving microbial transfer of wild mice into laboratory mice has demonstrated the role of the natural or wild microbiota in disease resistance and protective immune mechanisms (35). Further, a more recent study by the same group also showed that laboratory mice bred from such wild mice (referred to as wildlings) exhibited the elevated microbial diversity of the parent wild mice accompanied by a stability to perturbations such as antibiotics and diet, implying that a natural or wild immunity could be more comparable to that seen in the diversity of human illnesses (36). Hence, we considered it pertinent to include the bacterial taxa from some of these wild mouse samples (formerly wild wood mice [*Apodemus sylvaticus*] and wild Mus musculus *domesticus* mice) in the database, in addition to cultured oral isolates which could be used for *in vivo* and *in vitro* experimentations in the future. We fully appreciate that at present we report this from a limited number of sources and therefore representing only a fraction of the actual diversity observed in the wild, but we have plans to further expand this resource with the inclusion of more diverse sources based on availability.

It is also pertinent here to point out the presence of certain taxa in this murine oral microbial population potentially of intestinal origin, which could be attributed to the coprophagic behavior in murine populations. Compared to the mouse intestinal bacterial collection database, we notice the presence of some shared taxa with our database, particularly species belonging to the order *Lactobacillales* (19). Equally, oral bacteria might also be found in the gut, particularly lactobacilli because of their aciduric nature. However, despite this overlap, the oral microbiome in mice remains distinct, far simpler, and less diverse than in the murine gut microbiome (see Fig. S2 and S3), as we have demonstrated by amplicon sequencing and characterization of a limited subset of murine fecal samples.

10.1128/mSystems.01222-20.6 FIG S2 Beta-diversity analyses of the oral and gut microbiome of various mouse species. Beta diversity analyses of the oral and fecal microbial populations of SPF laboratory mice (*n* = 8) and the wood mouse *Apodemus sylvaticus* (*n* = 12) were carried out using the 16S rRNA gene amplicon community data targeting the V1-V2 region of the gene. The sequences were analyzed using the DADA2 v1.8 pipeline, and the assembled ASVs were assigned taxonomy using the SILVA rRNA reference database v138.1. Download FIG S2, TIF file, 1.5 MB. Copyright © 2021 Joseph et al. 2021 Joseph et al. This content is distributed under the terms of the Creative Commons Attribution 4.0 International license.

It is necessary to stress that this study primarily describes the development of a framework for this murine oral microbial database, which by its nature remains a work in progress. Work is under way with collaborators at the Forsyth Institute, Cambridge, MA, to make the final version of this database publicly available as the Mouse Oral Microbiome Database (MOMD), on the lines of the Human Oral Microbiome Database (HOMD) (16). This will enable the addition of sequences from other researchers in the field; these will be curated and made publicly available. Meanwhile, the cultured isolates analyzed for this database can be made available to interested researchers on request by contacting the authors. Further work is also being undertaken using sequence analysis and cloning to characterize the uncultured as well as the identified but unnamed novel species, including their taxonomic nomenclature, for the further expansion of the database.

We hope that this should enable researchers across the world to access and develop suitable reference data sets for both culture and culture independent studies of the murine oral microbiome.

## MATERIALS AND METHODS

### Mouse sampling and ethics.

All animal experiments were conducted in accredited facilities in accordance with the UK Animals (Scientific Procedures) Act 1986 (Home Office license number 7006844). Fieldwork for the wild Mus musculus *domesticus* work was approved by the Animal Welfare Ethical Review Body (AWERB) at the Department of Zoology, University of Oxford.

Conventional SPF C3H/Orl and BALB/c mice were maintained in individually ventilated cages (IVCs) at the animal care facilities of Queen Mary University of London. Conventional SPF C57BL/6J mice were maintained in IVCs at the animal care facilities of King’s College London. SPF C57BL/6J and CD-1 mice were also purchased commercially from Charles River Laboratories UK. In all, 191 SPF mice have been sampled over the course of 6 years for various experimental studies, and isolates obtained from the swabbing of these mice have been used for the analysis and development of this database.

One set (set 1) of formerly wild, now laboratory-bred, *A. sylvaticus* wood mice sampled in 2014 (*n* = 20) was originally established from wild wood mice that were live trapped from the UK woodlands and are now housed at the University of Edinburgh. Wild wood mice sampled in January 2017 (*n* = 39) were housed at the facilities at Fera Science (Sand Hutton, York), and the colony began with wild-caught wood mice from the grounds of the Institute; these animals had been laboratory-bred for 20 years and are referred to as set 2.

Wild house mice (Mus musculus *domesticus* [*n* = 21]) were sampled on the island of Skokholm in Wales, UK. These mice were captured temporarily in traps, sampled, and then released back into the wild.

### Bacterial culturing.

The murine oral cavity was swabbed for 30 s, using sterile fine-tip rayon swabs (VWR International), while the animal was held in a scruff. The swab was then placed in a tube containing 100 μl of reduced John’s transport medium (see Text S1 in the supplemental material). Swabs from wild mice were stored at −20°C before being transported on ice to the laboratory. Serial dilutions of the suspension were spread onto blood agar plates containing 5% defibrinated horse blood (TCS Biosciences, UK) and incubated for aerobic and anaerobic (80% N_2_, 10% H_2_, and 10% CO_2_) growth at 37°C for 48 h in a Don Whitley anaerobic chamber. The CFUs of predominant cultivable bacteria on each plate were counted. On an average, four to six different colony types could be identified on each blood agar plate. Every different colony morphology type observed was isolated and purified by subculture by restreaking the samples twice on fresh blood agar plates. Once the purity was established, the cultured isolate was cryopreserved for storage in Microbank bead tubes (Prolab Diagnostics) in duplicate. Briefly, a loopful of pure culture was aseptically transferred into the manufacturer’s cryopreservative liquid containing beads, the tube was inverted four to five times for emulsification and allowed to stand for 2 min. Any excess liquid was aseptically removed, and the bead tube was then frozen at −70°C.

10.1128/mSystems.01222-20.1 TEXT S1 Composition of John’s Transport medium. Download Text S1, DOCX file, 0.01 MB. Copyright © 2021 Joseph et al. 2021 Joseph et al. This content is distributed under the terms of the Creative Commons Attribution 4.0 International license.

### 16S rRNA gene amplification and sequencing.

Genomic DNA for each isolated bacterial strain was extracted by using a GenElute bacterial DNA kit (Sigma-Aldrich), following the Gram-positive protocol according to manufacturer’s instructions, and used as a template for PCR. The 16S rRNA gene in the bacterial strains was amplified using modified versions of the universal 27FYM and 1492R 16S rRNA gene primers with built-in redundancies (see Text S2), using Phusion Green Hot Start II High Fidelity PCR Master Mix (Thermo Fisher Scientific). PCR conditions were as follows: initial denaturation at 98°C for 30 s; followed by 25 cycles of 98°C for 10 s, 47°C for 45 s, and 72°C for 30 s; followed in turn by a final extension at 72°C for 10 min. The amplified products were purified using Macherey-Nagel NucleoSpin gel and PCR Clean-Up (Fisher Scientific), followed by Sanger sequencing using the universal M13 primers M13 uni(-21) and M13 rev(-29) (Eurofins Genomics). For certain samples, internal primers for the 16S rRNA gene (342R, 357F, 519R, 907R, 926F, 1100R, 1114F, and 1392R) were also used for sequencing to improve the sequence coverage accuracy. All primer sequences have been provided in Text S2.

10.1128/mSystems.01222-20.2 TEXT S2 Modified version of the universal primers of the 16S rRNA gene. Download Text S2, DOCX file, 0.01 MB. Copyright © 2021 Joseph et al. 2021 Joseph et al. This content is distributed under the terms of the Creative Commons Attribution 4.0 International license.

### Allocation of mouse oral taxa.

The forward and reverse sequences for each sample were assembled using the CAP3 assembly tool (37). Sequences were identified by BLAST interrogation of the NCBI nucleotide database. A sequence identity threshold of 98.5% was used for assignation to species, which is consistent with current recommendations (38) and the value used previously for the related human and canine oral databases (17, 18). Sequences were aligned by means of the CLUSTALW algorithm in Bioedit (39), and maximum-likelihood phylogenetic trees for each genus were constructed using MEGA7 (40) with 100 bootstrap replicates. Each species-level taxon was assigned an MOT number. For taxa that were not identified as validly proposed species at 98.5% identity, a novel species-level designation was assigned as “Genusname_species1,” and a new MOT number was allocated. For isolates that could not be definitively distinguished by 16S rRNA gene sequence analysis, each of the possible matching species was assigned a unique MOT ID in order to ensure maximum diversity capture. Assignment of taxa was also carried out using 16S rRNA gene amplicons selected from the MiSeq sequencing data that were not assigned an ID based on the existing reference database and then following the same BLAST protocol as described above. Visualization and annotation of the final phylogenetic tree of the identified 103 MOTs was performed using the web interface of iTOL v4 (41).

### MiSeq 16S rRNA gene sequencing library preparation and DNA sequence analysis.

Whole genomic DNA was extracted from the above swabs using the DNeasy PowerSoil kit (Qiagen) according to the manufacturer’s instructions. Cell lysis was performed in the PowerBead tubes provided with the kit by bead beating on a vortex at maximum speed for 20 min. PCRs were performed with Phusion Green Hot Start II High Fidelity PCR Master Mix (Thermo Scientific) targeting the V1-V2 variable regions of the 16S rRNA gene using fusion primers 27F-YM (AGAGTTTGATYMTGGCTCA) and 338R-R (TGCTGCCTCCCGTAGRAG) combined with MiSeq adaptors and barcodes to achieve a double indexing system. The PCR conditions were as follows: initial denaturation for 5 min at 95°C; followed by 25 cycles of 95°C for 45 s, 53°C for 45 s, and 72°C for 45 s; followed in turn by a final extension of 72°C for 5 min. The amplified PCR products were cleaned and normalized in equimolar amounts by using a Sequal Prep normalization plate kit (Thermo Fisher Scientific). Extraction kit controls and PCR negative controls were included in the amplification plates, as well as sequencing pools. Pooled amplicons were sequenced at the Barts and the London Genome Centre using an Illumina MiSeq 2 × 250 flow cell for paired-end sequencing. The generated reads were quality checked, filtered, trimmed, denoised, dereplicated, and assembled into amplicon sequence variants (ASVs) using the DADA2 v1.8 pipeline (42). The assembled ASVs were then assigned taxonomy at the genus and species level using a custom-formatted reference database constructed using the taxa included in this database. Compared to the mean of total reads in the murine swab samples, the negative controls generated a very low percentage of reads (<0.1% in the PCR control and <1% in the kit control). Once this was confirmed, the negative samples were eliminated from further analyses. The generated ASV counts were normalized for sequencing depth by using the median of ratios method in the DeSeq2 (43) package in R, followed by beta-diversity and relative abundance analyses of the microbial population. Graphical analysis and plots were created using the R packages phyloseq (44) and ggplot2 (45). The raw sequencing reads have been uploaded to the NCBI SRA database (accession no. PRJNA642845).

### Bacterial genome sequencing.

A selection of bacterial isolates from the database were cultured, purity checked, and genomic DNA extracted using the GenElute Bacterial Genomic DNA kit (Sigma-Aldrich). These isolates are representatives of the cultured microbial population observed in various murine studies and include multiple candidates of the more commonly observed *Streptococcus*, *Lactobacillus*, and *Staphylococcus* species-level taxa. Genomic DNA libraries were prepared by MicrobesNG UK using Nextera XT Library Prep kit (Illumina, San Diego, CA) and sequenced on the Illumina HiSeq using a 250-bp paired-end protocol. Reads were adapter trimmed using Trimmomatic 0.30 with a sliding window quality cutoff of Q15 (46). *De novo* assembly was performed on samples using SPAdes version 3.7 (47), and contigs were annotated using Prokka 1.11 (48). Further genome analysis and annotation was also performed using the RAST server (https://rast.nmpdr.org/) (49). Phylogenetic distance and relatedness of certain isolates were determined using the genome-to-genome distance calculator, in the form of an *in silico* DNA-DNA hybridization (50).

### Data availability.

The 16S rRNA gene V1-V2 region amplicon sequencing data from this study are available from the NCBI SRA database under accession no. PRJNA642845. Draft genome sequences of representative murine oral bacterial isolates are available to download from NCBI BioProject no. PRJNA671681. 16S rRNA gene sequences of the novel, unnamed bacterial isolates from this study are available under NCBI accession numbers MN095260 to MN095271. The 16S rRNA gene sequences for all the isolates analyzed in this study are available under NCBI accession numbers MW175535 to MW175859. Custom taxonomy reference data sets of the database for NGS analysis using mothur and DADA2 pipelines are available to download from https://figshare.com/s/2470f05ab77cdf40b2f8.

10.1128/mSystems.01222-20.3 TEXT S3 Internal primers of the 16S rRNA gene used for primer walking. Download Text S3, DOCX file, 0.01 MB. Copyright © 2021 Joseph et al. 2021 Joseph et al. This content is distributed under the terms of the Creative Commons Attribution 4.0 International license.

10.1128/mSystems.01222-20.4 TEXT S4 MiSeq 16S rRNA gene sequencing library preparation and DNA sequence analysis of murine fecal samples. Download Text S4, DOCX file, 0.01 MB. Copyright © 2021 Joseph et al. 2021 Joseph et al. This content is distributed under the terms of the Creative Commons Attribution 4.0 International license.

10.1128/mSystems.01222-20.7 FIG S3 Relative abundances in the gut microbiome of various mouse species. The relative abundances of the fecal microbial population in SPF laboratory mice (C3H/Orl and C57BL/6J) (A) and the formerly wild wood mouse, *Apodemus sylvaticus* (B), sampled from two sources (set 1 and set 2), were analyzed using Illumina MiSeq sequencing technology by amplifying the V1-V2 region of the 16S rRNA gene sequences. The amplicon sequencing data were analyzed using the DADA2 v1.8 pipeline, and the assembled ASVs were assigned taxonomy using the SILVA rRNA reference database v138.1. The top 10 most relatively abundant taxa are listed. Download FIG S3, TIF file, 2.7 MB. Copyright © 2021 Joseph et al. 2021 Joseph et al. This content is distributed under the terms of the Creative Commons Attribution 4.0 International license.
