# Supplementary figures and images for: A 16S rRNA Gene and Draft Genome Database for the Murine Oral Bacterial Community
Source: mSystems. 2021 Feb 9;6(1):e01222-20. doi: 10.1128/mSystems.01222-20 (PMC7883545; doi:10.1128/mSystems.01222-20)

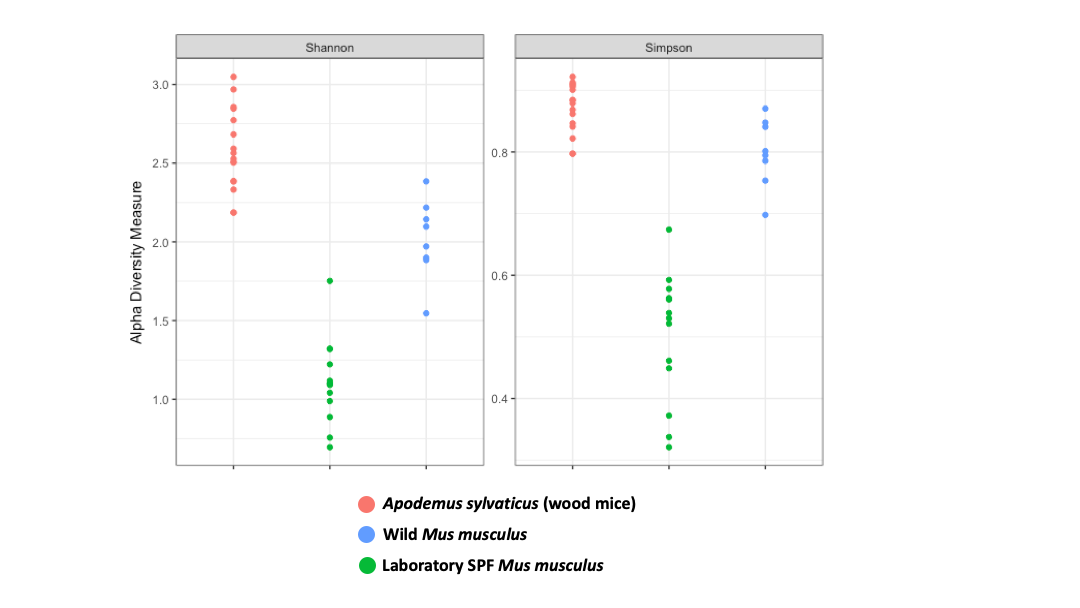

Supplement: FIG S1 [file mSystems.01222-20-sf001.tif]

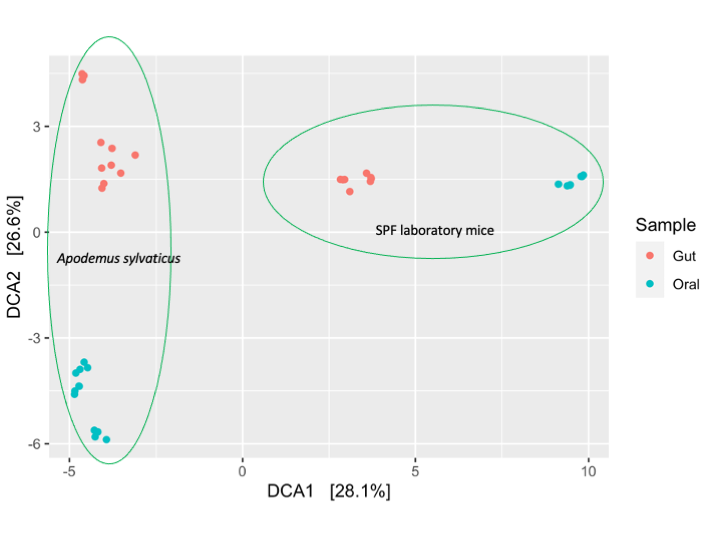

Supplement: FIG S2 [file mSystems.01222-20-sf002.tif]

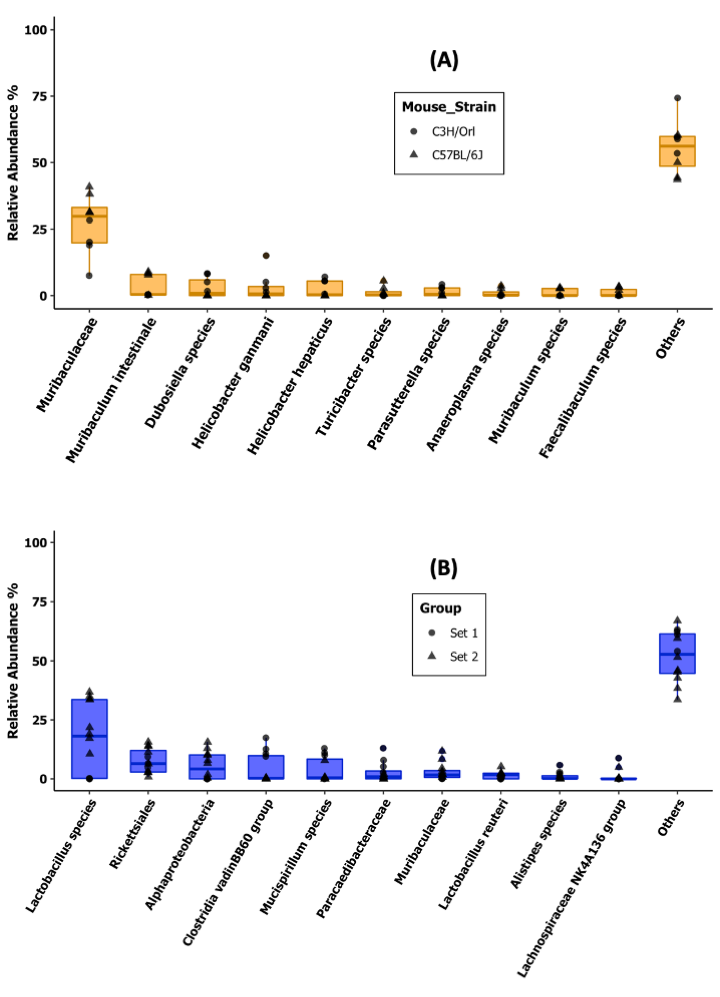

Supplement: FIG S3 [file mSystems.01222-20-sf003.tif]
