# Supplementary material for: A 16S rRNA Gene and Draft Genome Database for the Murine Oral Bacterial Community
Source: mSystems. 2021 Feb 9;6(1):e01222-20. doi: 10.1128/mSystems.01222-20 (PMC7883545; doi:10.1128/mSystems.01222-20)
Supplement: TEXT S1 [file mSystems.01222-20-s0001.docx]

**Supplementary Text 1. Composition of John’s Transport medium**

**g/L**

Yeast Extract 5

Proteose Peptone 1

Cysteine-HCl 0.5

Sodium chloride NaCl 8.5

Disodium hydrogen phosphate Na_2_HPO_4_ 0.868

Potassium dihydrogen phosphate KH_2_PO_4_ 0.528

Tween-80 1 ml

Glycerol 150 ml

Distilled water 800 ml

The components were dissolved, pH adjusted to 7.0 with 1M NaOH solution and final volume made up with distilled water to 1 L, followed by sterilisation by autoclaving.
