# Supplementary material for: A 16S rRNA Gene and Draft Genome Database for the Murine Oral Bacterial Community
Source: mSystems. 2021 Feb 9;6(1):e01222-20. doi: 10.1128/mSystems.01222-20 (PMC7883545; doi:10.1128/mSystems.01222-20)
Supplement: TEXT S2 [file mSystems.01222-20-s0002.docx]

**Supplementary Text 2. Modified version of the universal primers of the 16S rRNA gene**

**BLIZF2:** GTAAAACGACGGCCAGTGATCAGAGTTTGATYMTGGCTCAG

**BLIZR2:** CAGGAAACAGCTATGACCATATTACCTTGTTAYGACTT
