# Supplementary material for: A 16S rRNA Gene and Draft Genome Database for the Murine Oral Bacterial Community
Source: mSystems. 2021 Feb 9;6(1):e01222-20. doi: 10.1128/mSystems.01222-20 (PMC7883545; doi:10.1128/mSystems.01222-20)
Supplement: TEXT S3 [file mSystems.01222-20-s0003.docx]

**Supplementary Text 3. Internal primers of the 16S rRNA gene used for primer walking**

**27f** 5'AGAGTTTGATCMTGGCTCAG>

**342r** 5'CTGCTGCSYCCCGTAG>

**357f** 5'CTCCTACGGGAGGCAGCAG>

**519r** 5'GWATTACCGCGGCKGCTG>

**907r** 5'CCGTCAATTCMTTTRAGTTT>

**926F** 5'AAACTYAAAKGAATTGACGG>

**1100r** 5'GGGTTGCGCTCGTTG>

**1114f** 5'GCAACGAGCGCAACCC>

**1392r** 5'ACGGGCGGTGTGTRC>

**1492r** 5'TACGGYTACCTTGTTACGACTT>

**1525r** 5'AAGGAGGTGWTCCARCC>
