# Supplementary material for: A 16S rRNA Gene and Draft Genome Database for the Murine Oral Bacterial Community
Source: mSystems. 2021 Feb 9;6(1):e01222-20. doi: 10.1128/mSystems.01222-20 (PMC7883545; doi:10.1128/mSystems.01222-20)
Supplement: TEXT S4 [file mSystems.01222-20-s0004.docx]

**Supplementary Text 4. MiSeq 16S rRNA gene sequencing library preparation and DNA sequence analysis of murine fecal samples**

Fresh fecal pellets were collected using sterile forceps and frozen at -80^o^C on the day of collection. Genomic DNA extraction and 16S rRNA gene library preparation and sequencing were performed as described for the oral swabs. The generated reads were quality checked, filtered, trimmed, denoised, dereplicated and assembled into amplicon sequence variants (ASVs) using the DADA2 v1.8 pipeline. The assembled ASVs were then assigned taxonomy at the genus and species level using the SILVA rRNA database version 138.1. The generated ASV counts were normalized for sequencing depth using the median of ratios method in the DeSeq2 package in R, followed by beta diversity and relative abundance analyses of the microbial population. Graphical analysis and plots were created using the R packages phyloseq and ggplot2. The raw sequencing reads have been uploaded to the NCBI SRA database Bioproject Accesion No. PRJNA679590.
